# Rethinking Cefepime Neurotoxicity: An Integrated PK–Neuromonitoring Approach for Neurocritical Care

**DOI:** 10.1007/s12028-026-02544-w

**Published:** 2026-05-23

**Authors:** Helena Barrasa, Thiago H. G. Silva, Ana Alarcia-Lacalle, Ana Tejero, Amaia Quintano

**Affiliations:** 1 Osakidetza Basque Health Service, Araba University Hospital, Intensive Care Unit, 01009 Vitoria-Gasteiz, Spain; 2 Bioaraba, Care and Technology Applied to Critical and Post-Critical Patients Research Group, Vitoria-Gasteiz, Spain; 3 Pharmacokinetic, Nanotechnology and Gene Therapy Group (Pharma Nano Gene), Centro de Investigación Lascaray Ikergunea, Faculty of Pharmacy, University of the Basque Country UPV/EHU, 01006 Vitoria‐Gasteiz, Spain

**Keywords:** (MeSH): Cefepime, Neurotoxicity syndromes, Encephalopathy, Electroencephalography, Therapeutic drug monitoring, Critical care, Anti-bacterial agents

## Abstract

Cefepime-induced neurotoxicity (CIN) remains under-recognized despite being a largely exposure-driven and multifactorial complication in neurocritical care. Early identification is often hindered by limited clinical evaluability, delayed electroencephalography (EEG) access, and rapidly evolving renal function that compromises dosing accuracy. Although higher cefepime exposure is consistently associated with increased neurotoxicity risk, no universally accepted therapeutic range clearly separates efficacy from toxicity across critically ill populations. This Viewpoint proposes an anticipatory approach integrating pharmacokinetic principles, therapeutic drug monitoring (TDM), and simplified neuromonitoring to facilitate earlier detection and prevent progression to severe encephalopathy or nonconvulsive seizures. We outline a pragmatic framework to enhance neurological safety while preserving antimicrobial efficacy.

## Introduction

Cefepime is widely used in neurocritical care owing to its broad-spectrum activity and reliable penetration into the central nervous system when inflammation is present [1, 2]. Yet its neurotoxic potential remains systematically underestimated. Cefepime-induced neurotoxicity (CIN) typically presents as an acute, fluctuating encephalopathy—often accompanied by inattention, confusion, myoclonus, or aphasia—and emerges within the first days of therapy [1, 3]. Early neurological changes are frequently obscured by sedation or critical illness; in other patients, symptoms are attributed to more common encephalopathies such as sepsis, metabolic disturbances, or baseline brain injury[3–5]. As a result, cefepime is often continued longer than it should be, delaying the single most effective intervention: withdrawing the drug.

Notably, CIN is not an idiosyncratic phenomenon. It is largely exposure-driven and therefore potentially preventable, but its clinical expression is multifactorial and extends beyond isolated drug accumulation. Impaired or unstable renal function, preexisting neurological disease, white-matter abnormalities, advanced age, higher body weight, and broader medical comorbidity appear to increase vulnerability [2, 6]. Because neither drug exposure nor early neurophysiological changes are systematically assessed in routine care, recognition is often delayed and dependent on overt clinical deterioration [6, 7].

In this Viewpoint, we argue for a shift from reactive recognition to an anticipatory approach. We propose a pragmatic framework that integrates early identification of vulnerable patients, simplified neuromonitoring, and timely therapeutic drug monitoring (TDM) to detect harmful exposure before severe neurological injury develops.

## Clinical Spectrum and Diagnostic Pitfalls

The clinical expression of CIN is recognizable yet deceptively nonspecific. Across multiple cohorts, encephalopathy is the dominant presentation (70–90%), typically arising within 2–5 days of therapy [3, 5]. Patients may show fluctuating awareness, inattention, or disorientation resembling sepsis-associated delirium. Focal manifestations such as myoclonus or aphasia are common [6]. While CIN can resemble septic or other toxic-metabolic encephalopathies, it often coexists with these conditions, given that cefepime is frequently deployed in patients with severe infection. Although often perceived as uncommon, CIN occurs in up to 20% of critically ill patients[5], yet this estimate is largely derived from general ICU cohorts and may not reflect the true burden in the neurocritically ill.

In patients who are not fully evaluable, early neurological changes remain obscured [3], and inconsistent use of structured delirium assessments (e.g., CAM-ICU) may further delay recognition—until more advanced dysfunction appears and drug accumulation has already compounded neurological injury.

Diagnostic misattribution remains a major pitfall. Early CIN manifestations overlap almost perfectly with the differential diagnosis of neurocritical care: sepsis-associated encephalopathy, uremic or hepatic dysfunction, sedative effects, metabolic disturbances, and preexisting brain injury [2, 8]. Myoclonus may be dismissed as metabolic, aphasia attributed to prior stroke, and fluctuating responsiveness mistaken for delirium. This overlap explains why early suspicion of CIN is uncommon, even when a clear temporal association with cefepime exists.

Electroencephalography (EEG) findings reinforce this pattern. When obtained, EEG often reveals toxic–metabolic patterns, including periodic discharges [6, 9]. Triphasic waves may also be present. However, cefepime-related EEG abnormalities are more commonly characterized by periodic discharges with a prominent initial negative phase, whereas classic triphasic waves typically exhibit a smaller first wave[9]. Recognizing this neurophysiologic distinction is important, lest practitioners discontinue cefepime on the basis of potentially unrelated encephalopathic findings. Yet these findings are also common in other encephalopathies, and without early suspicion, they are seldom interpreted as drug-related. As a result, EEG is frequently pursued only after substantial neurological deterioration.

Together, these features create an environment in which CIN is not distinguished, not suspected, and therefore not acted upon—allowing preventable neurotoxicity to progress. Recognizing the frequency, timing, and variability of these manifestations is essential to embedding CIN into the routine differential diagnosis of encephalopathy.

## Neuromonitoring: The Earliest Window of Detection

EEG provides the earliest objective evidence of evolving cerebral dysfunction, and in the minority of cases in which EEG is obtained sufficiently early, abnormalities appear hours to nearly a day before overt neurological deterioration. Early findings include diffuse slowing, loss of reactivity, and the evolution toward periodic discharges—patterns that precede the appearance of triphasic waves or nonconvulsive seizures[10].

In real-world practice, however, early EEG acquisition is uncommon. Continuous EEG is not universally available, and even in well-resourced units, competing priorities, staffing constraints, and technical limitations often delay monitoring[11]. For patients who are sedated, ventilated, or otherwise nonevaluable, this delay is especially consequential: electrophysiology may be the only accessible signal that CIN is emerging.

Simplified neuromonitoring systems, particularly bilateral frontal platforms such as BIS™ and SedLine®, are widely available and attractive when EEG is delayed. Although these devices are not diagnostic—their indices reflect algorithmic processing of frontal cortical signals rather than direct neurophysiological measurement—and are susceptible to confounders such as sedation, metabolic disturbances, and EMG activity, they may still highlight unexpected changes in cortical activity and help bridge delays in EEG availability [11, 12].

Observational data suggest that unexpected fluctuations may reflect evolving neurotoxicity[11]. In one illustrative patient receiving cefepime 2 g every 8 h at full dose—without adjustment despite the development of acute kidney injury with creatinine clearance approximately 30 mL/min—with suspected acute encephalopathy, SedLine monitoring showed early generalized slowing with recurrent periodic-appearing patterns despite absent sedation. These abnormalities prompted EEG confirmation of ictal-encephalopathic activity and TDM evidence of marked cefepime accumulation.

When used as triggers rather than diagnostic tools, simplified neuromonitoring systems can enhance early detection, particularly in nonevaluable patients, and support a shift from reactive recognition to anticipatory detection.

### PK, TDM, and Exposure-Based Dosing: Why Measurement Matters

CIN is an exposure-dependent complication, and in critically ill patients this exposure is shaped by well-recognized physiological alterations. Fluctuations in renal perfusion and glomerular filtration drive rapid shifts between augmented and reduced clearance; increases in extracellular volume alter the apparent volume of distribution; serum creatinine becomes unreliable in nonsteady-state conditions and acute kidney injury may coexist with uremia and the accumulation of other neuroactive solutes that can amplify neurological dysfunction[3, 5, 7]. Once in the CNS, cefepime is thought to impair GABA-mediated inhibition and promote cortical hyperexcitability, a mechanism that aligns with the prominence of myoclonus, periodic discharges, and nonconvulsive seizures observed in CIN[3, 6]. These changes make cefepime exposure difficult to predict from estimated renal function alone. Across studies, concentration thresholds vary due to differences in sampling strategies and analytical methods, yet consensus documents provide a pragmatic framework. Trough concentrations of approximately 8–16 mg/L have been proposed for antipseudomonal target attainment [13], whereas trough concentrations > 20 mg/L are associated with a substantially increased risk of neurotoxicity, although with limited discriminative accuracy, and values above 30–35 mg/L align with significant accumulation and a high likelihood of neurological injury. These ranges are not rigid cutoffs, but they offer clinically meaningful anchors for interpreting exposure. In continuous-infusion regimens, recent data indicate a higher toxicity threshold, with steady-state concentrations (Css) above ~ 60 mg/L associated with an increased risk of CIN[5, 14]. Importantly, these efficacy- and toxicity-associated ranges may overlap clinically, and no universally accepted therapeutic range currently exists for cefepime across critically ill populations, infusion strategies, and microbiological contexts.

Even appropriately adjusted regimens may lead to accumulation owing to rapidly changing physiology [1, 3, 9]. In this context, exposure must be measured, not inferred.

TDM can help close this gap, though its clinical value hinges on timing and interpretation, which differ for efficacy and toxicity. For efficacy, concentrations should be measured early, ideally within the first 24–48 h, to ensure target attainment at a time when bacterial killing is most critical[15, 16]. Toxicity, in contrast, reflects subacute accumulation that typically develops after several days of therapy, especially in the presence of unstable renal function. TDM should therefore be conceptualized as part of a trajectory, not a single value: early trough concentrations guide dose optimization, while follow-up trough concentrations—or steady-state concentrations in continuous infusion— detect creeping accumulation before neurological injury occurs [17, 18]. A practical advantage of cefepime is that clinically significant drug–drug interactions are relatively limited, which may reduce confounding in TDM interpretation compared with more interaction-prone agents.

Extended or continuous infusions may also offer a pharmacokinetic advantage in this context. By minimizing peak–trough variability and sustaining unbound concentrations above therapeutic thresholds, these strategies can achieve PK/PD targets, defined as maintaining free drug concentrations above the minimum inhibitory concentration for an adequate time fraction (fT > MIC), with lower total daily doses than traditional intermittent dosing—supporting a shift toward optimized therapy that balances dose, duration, and delivery to preserve efficacy while minimizing neurotoxicity risk[14]. These approaches do not eliminate the need for monitoring, but they can mitigate the unpredictability inherent in intermittent regimens, particularly when renal function is fluctuating.

This shift is essential to prevent neurotoxicity while preserving antimicrobial efficacy. In practice, however, the clinical utility of TDM is often limited by delayed analytical turnaround, cost, and restricted availability, requiring prioritization in the highest-risk patients[7]. Effective adoption also requires integration into antimicrobial stewardship workflows and a shift toward proactive, exposure-informed clinical practice.

### Extremes of Renal Clearance: ARC and RRT

The pharmacokinetic unpredictability of cefepime is magnified at the extremes of renal function, particularly in augmented renal clearance (ARC) and in patients receiving renal replacement therapy (RRT). In ARC, standard dosing may result in subtherapeutic concentrations and reduced target attainment[13]. Conversely, ARC does not confer absolute protection against CIN, because rapid physiological shifts may still lead to unexpected overexposure. In patients receiving continuous RRT, cefepime is readily cleared, and extracorporeal clearance is strongly influenced by modality and effluent flow[13]. Maintenance exposure remains difficult to predict, particularly with changing residual renal function. In both settings, exposure may be missed in either direction—underexposure or accumulation—supporting individualized dosing and TDM when feasible.

## A Practical Integrated Framework

Preventing CIN requires translating well-established pharmacological principles into a consistent bedside strategy. Because exposure fluctuates rapidly in critical illness, an anticipatory approach combining clinical assessment, real-time renal evaluation, therapeutic drug monitoring, and neuromonitoring offers a pragmatic way to detect harmful trajectories before overt neurological injury develops.

### Early Risk Stratification: Identifying Vulnerable Patients

Risk of CIN is greatest in those with impaired or unstable renal function, hypoalbuminemia, advanced age, higher body weight, preexisting neurological vulnerability, or those receiving high-dose cefepime. The challenge is amplified when early neurological assessment is limited by sedation, mechanical ventilation, or baseline encephalopathy. Patients with ARC or RRT should be considered high risk owing to unpredictable exposure. Identifying these patients early clarifies where closer surveillance and exposure-based dosing may have the greatest impact[2, 5].

### Measure or Refine Estimates: Real-Time Renal Function Matters

Measured creatinine clearance—derived from timed urine collections—offers a more accurate assessment of real-time renal function and can meaningfully inform initial dosing and subsequent adjustments in high-risk patients[19]. When available, cystatin C may complement creatinine-based assessment, and combined creatinine–cystatin C estimates may better reflect renal function and predict the clearance of renally eliminated drugs than creatinine alone[20, 21]. However, cystatin C should be viewed as a promising adjunct rather than a fully established standard in the ICU, since implementation remains limited by cost, turnaround time, and local availability.

### Exposure-Based Dosing: Integrating Early and Follow-Up TDM

TDM complements clinical judgment when interpreted longitudinally[16, 17]. Early TDM (24–48 h) primarily ensures therapeutic exposure in the critical initial phase of treatment, while follow-up TDM supports both efficacy and safety by detecting accumulating exposure—the key driver of CIN, particularly when renal function is evolving. Viewing cefepime concentrations as a trend rather than isolated values allows proactive dose adaptation.

### Dosing Strategy: When Extended or Continuous Infusion May Help

In patients at heightened risk of CIN, extended or continuous infusion can offer a more stable pharmacokinetic profile. By reducing concentration fluctuations, these regimens may achieve PK/PD targets with lower total daily doses, supporting the principle of attaining the minimum effective exposure while minimizing the risk of accumulation. Continuous infusion does not replace monitoring but may provide a safer baseline from which to individualize therapy[14].

### Clinical Suspicion and Proactive Surveillance

A high index of suspicion remains essential in all patients receiving cefepime—whether they are clinically evaluable or not. In evaluable patients, disproportionate inattention, fluctuating responsiveness, new aphasia, or myoclonus should prompt consideration of evolving CIN. In nonevaluable or high-risk patients, a proactive surveillance strategy is needed. Incorporating safety-focused TDM with simplified neuromonitoring (e.g., BIS/SedLine) can surface early cortical changes that precede overt encephalopathy. When such signals arise—or when concentrations indicate accumulating exposure—urgent EEG and reassessment of dosing may prevent progression to more severe CIN[11, 22, 23].

### Antimicrobial Selection as Prevention

In selected high-risk patients with non-CNS infections, the simplest preventive intervention may be choosing an alternative equally effective antibiotic when microbiologically appropriate[24]. This does not diminish cefepime’s value but acknowledges that stewardship and neurological safety may occasionally align.

### An Integrated Workflow

**Fig. 1:**
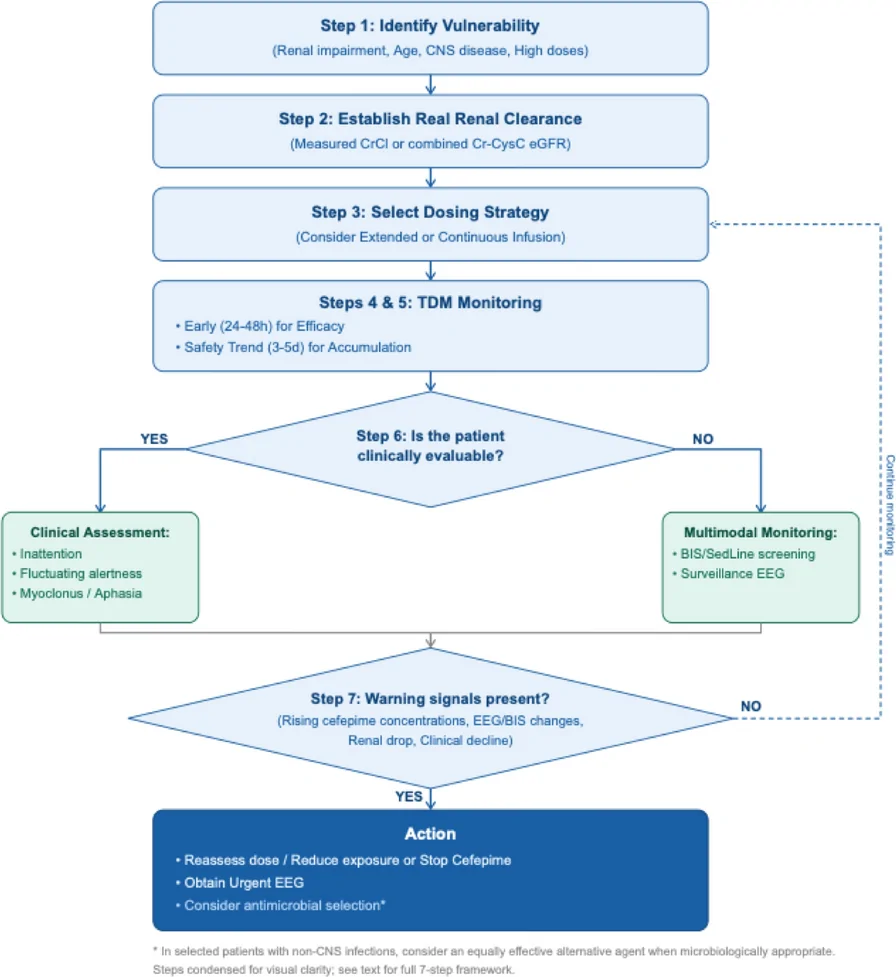
Anticipatory CIN prevention decision tree. A stepwise bedside strategy integrating early risk identification, renal function assessment, exposure-based dosing, therapeutic drug monitoring, and neuromonitoring to facilitate proactive detection of cefepime-induced neurotoxicity

A pragmatic approach may follow the sequence below and is summarized in Fig. 1:

1. Identify risk on the basis of renal vulnerability, neurological susceptibility, and evaluability.
2. Measure renal clearance when appropriate.
3. Select the dosing strategy, including extended or continuous infusion.
4. Obtain early TDM to confirm therapeutic exposure.
5. Repeat TDM to detect emerging accumulation.
6. Use neuromonitoring when clinical evaluation is unreliable.
7. Act promptly when clinical, pharmacokinetic, or neurophysiological warning signals appear.

This integrated workflow operationalizes an anticipatory approach that complements, rather than replaces, clinical judgment.

## Future Directions

As noted above, current approaches to TDM are constrained by delayed turnaround times, limited availability outside daytime hours, and workflows dependent on batch processing rather than real-time clinical need. These operational barriers mean that TDM often arrives too late to guide anticipatory adjustment of cefepime exposure, particularly in patients whose renal function and drug levels can shift over hours. Closing this temporal gap will require rapid or point-of-care β-lactam assays, ideally with reliable estimation of free concentrations and validation in critically ill populations.

Beyond faster measurements, model-informed precision dosing (MIPD)—potentially enhanced by machine-learning approaches that integrate evolving renal function, drug levels, and patient-specific characteristics—may help forecast exposure trajectories rather than merely react to them[25, 26]. Parallel advances in neuromonitoring could provide complementary early-warning signals: integrating simplified bilateral monitors with automated pattern detection, or correlating their dynamics with early EEG changes, may help identify cortical instability before overt encephalopathy develops.

Future research should link real-time exposure, renal function, and neurophysiological signatures to refine actionable thresholds and optimize dosing strategies, including continuous infusion. Ultimately, prospective interventional trials are needed to determine whether an anticipatory, multimodal framework can reduce CIN in vulnerable patients.

## Conclusions

CIN is a largely exposure-driven complication that remains potentially preventable, but its development is more complex than any single threshold can capture. In neurocritical care—where neurological evaluation is often limited and renal function evolves rapidly—relying on delayed clinical deterioration risks missing the narrow window in which toxicity can be averted. An anticipatory strategy that identifies vulnerable patients, measures true renal clearance, tailors exposure, and leverages neuromonitoring as an early warning system aligns bedside practice with the pharmacological and neurophysiological realities that drive CIN. By shifting from retrospective recognition to proactive detection, clinicians can intervene earlier, protect neurological function, and preserve the antimicrobial value of cefepime.
